# The size of the Hill Sachs defect increases during reduction of a first time shoulder dislocation in older adults: a pilot study in 20 cases

**DOI:** 10.1186/s40634-023-00667-z

**Published:** 2023-12-22

**Authors:** Miguel Angel Ruiz Ibán, Roque Pérez Expósito, Agustina Vicente Bártulos, Rosa Vega Rodriguez, Raquel Ruiz Díaz, Jorge Diaz Heredia

**Affiliations:** 1 Shoulder and Elbow Unit, Orthopaedic Surgery and Trauma Service, Hospital Universitario Ramón y Cajal, Cta. Colmenar Km 9,100, 28046 Madrid, Spain; 2 Present Address: Departamento de Cirugía, Ciencias Sanitarias Y Medicosociales, Universidad de Alcalá de Henares, Madrid, Spain; 3 Área De Traumatología y Ortopedia, Universidad CEU San Pablo, Madrid, Spain; 4 Radiology Service, Hospital Universitario Ramón y Cajal, Madrid, Spain

**Keywords:** Shoulder, Shoulder instability, Hill-Sachs defect, Reduction, First time shoulder dislocation, Shoulder dislocation

## Abstract

**Purpose:**

To evaluate if the size of Humeral Hill-Sachs Defects (HSDs) increases during reduction in the emergency department (ED) in subjects that have a first-time anterior shoulder dislocation.

**Methods:**

Subjects more than 18 years old presenting to the ED a first-time anterior shoulder dislocation were included. A computed tomography was performed prior to any reduction attempt (Pre-CT). The shoulder was reduced in the emergency room with intraarticular lidocaine; if two attempts failed, the shoulder was reduced under anaesthesia. A second CT was performed after reduction of the shoulder (Post-CT).

CT were evaluated using the Osirix software. A 3-dimensional reconstruction of the humeral head was performed and the maximum width of the humeral defect, maximum depth of the humeral defect and total volume of the humeral defect were measured. The relative increase in size was calculated.

**Results:**

Twenty subjects were included in the study. All subjects presented HSDs in the Pre-CT that had a width of a median of 9.9(interquartile range:2.9)mm, a depth of 7.0(3.0]mm and a volume of 355(333)mm^2^. The HSD in the Post-CT had a width of 10.9(3.0)mm (an increase of 7.23[8.5]%, significant differences, *p* = 0.0001) a depth of 7.2(2.7)mm (an increase of 9.93[20.7]%, significant differences, *p* < 0.0001) and a volume of 469(271) mm2 (an increase of 27.5[26.9]%, significant differences, *p* < 0.0001). There were size increases larger than 25% in 15/20 (75%) of subjects.

**Conclusion:**

Standard reduction manoeuvres performed in a first-time anterior shoulder dislocation increase the size of the HSD. This increase in size is larger than 25% in four out of five cases.

**Level of evidence:**

IV, prospective cases series study.

## Introduction

The Hill-Sachs defect (HSD) is a humeral bone defect described initially by Malgaine [14] and latter by Hill and Sachs [11] that is found in subjects who have a anterior shoulder dislocation. It is found in up to 95% to 100% of subjects with anterior recurrent shoulder instability [13, 19] and in 58% to 93% of subjects with a first-time anterior shoulder dislocation [13, 16]. The size and shape of HSD are strongly associated with the incidence of recurrence of the instability [15] and with the chances of failure after surgical treatment [2].

The HSD develops as the softer trabecular bone of the head of the humerus impacts with the hard anteroinferior glenoid bone when the shoulder dislocates. It is not well understood when exactly the bone defect develops. Classically it was considered that the lesion develops at the moment of the dislocation, as the head dislocates and impacts violently the anterior glenoid rim, with the arm in abduction and external rotation [1, 11, 19]. An elegant study by Kawakami et al. [12] showed that the defect does not develop with the arm in those extreme positions but in the mid-range position of the arm, and grows in internal rotation and abduction, suggesting a delayed development of the defect. This has been supported by some cadaveric studies [9] and is in line with research indicating that increasing the time the shoulder is kept dislocated, increases the size of the HSD [7, 10].

We hypothesized that the HSD does not have only a delayed onset, but that its size might increase during reduction of the dislocation. The aim of this study was to find out if HSD increases in size during the reduction manoeuvres of the shoulder.

## Material and methods

This was a longitudinal prospective case series study. The study was performed in the Emergency Department (ED) of a large university hospital.

The inclusion criteria for the participants were: (1) 18 years old or older, (2) To present in the ED with a first episode of anterior shoulder dislocation.

The exclusion criteria were: (1) that the participant had suffered any previous episode of dislocation or instability in the ipsilateral shoulder, (2) that the participant had suffered any previous surgical insult to the ipsilateral shoulder, (3) that, during the CT evaluation of the shoulder, any associated fracture (other than a HSD itself or an anterior rim glenoid lesion) was found, or (4) that the subject was incapable to give consent to participate in the study.

All the subjects with a suspected anterior shoulder dislocation that entered the ED were initially assessed by the orthopaedic resident on-call. If, after proper anamnesis and full physical exam, suspicion of a first-time shoulder dislocation was established, the subject was offered to participate in the study. After informed consent was obtained, a computed tomography (CT) was immediately performed prior to any reduction attempt (Pre-CT).

After CT confirmation of the anterior dislocation and the absence of associated fractures, reduction of the shoulder was attempted using the following protocol: First, an intraarticular injection of 10 cm^3^ of mepivacaine 1% was performed through a lateral approach [18]. Then a reduction attempt was performed by the orthopaedic surgery resident using the traction/contra-traction manoeuvre [3], the Milch [18] or the FARES method [20]. If, after two attempts, reduction was not obtained, the participant was brought to the surgical theatre and reduction was obtained under deep sedation with propofol (initial bolus of 2 mg/kg, followed by perfusion of 6 mg/kg/h). Ventilatory assistance was provided as needed by an anaesthesiologist.

When reduction was obtained, a neurovascular exam was performed again, and the subject was immobilized in a standard sling in internal rotation for 4 weeks. Reduction was confirmed with a simple x-ray (anteroposterior and axillary view). A second CT (Post-CT) was performed in the following 4 weeks and before sling removal.

**Fig. 1:**
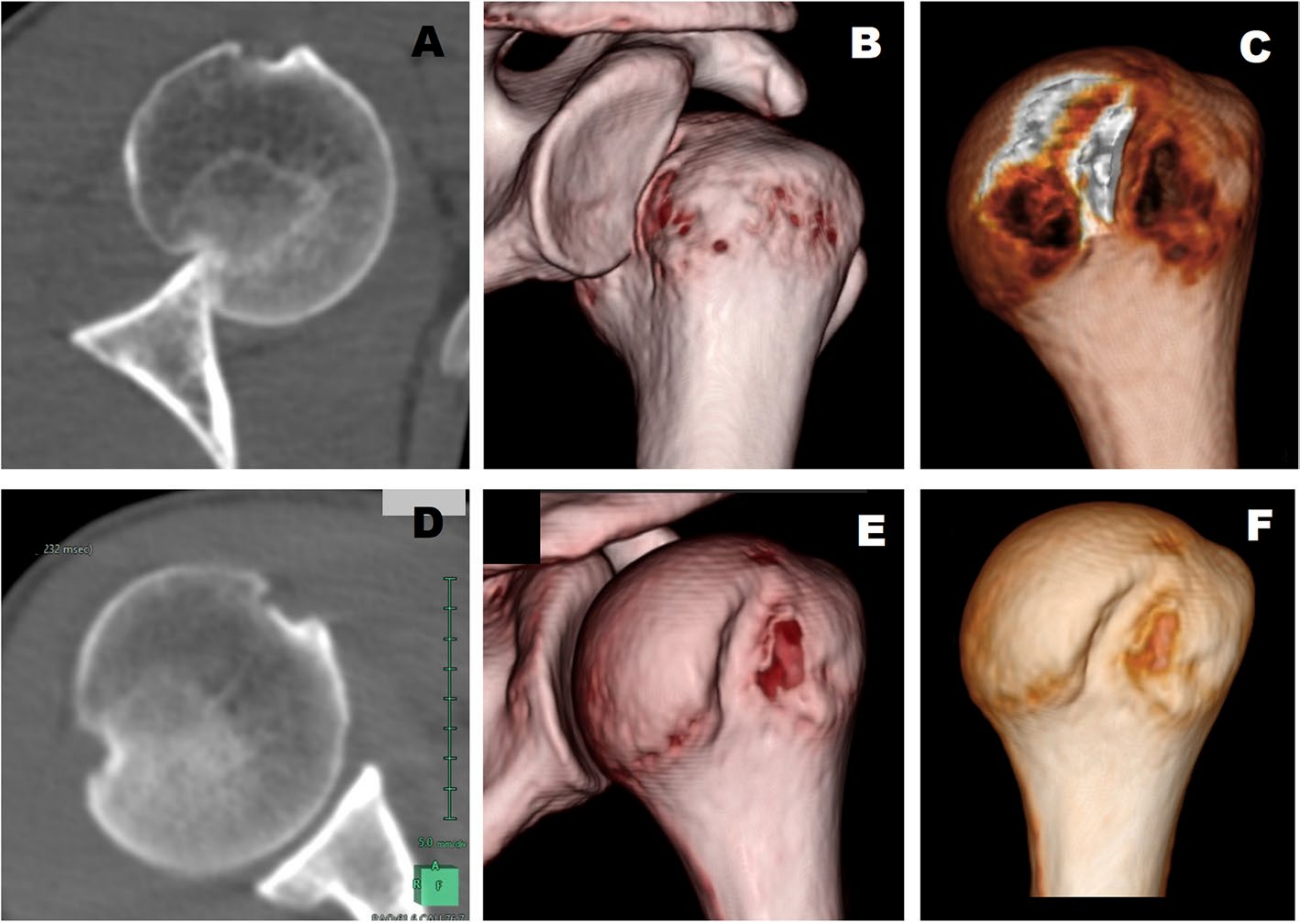
Prereduction and postreduction CT scans of one of the cases (Case 8 in Fig. 2). The prereduction images (**A**, **B** and **C**) show the dislocated shoulder and the small Hill- Sachs lesion. Postreduction CT images (**D**, **E** and **F**) show a moderate deepening of the defect

The primary outcome was the variation in the size of the HSDs between both CT (Fig. 1). The CT DICOM data was anonymized and imported to OsiriX DICOM viewer for Mac (version 11, Pixmeo, Geneva, Switzerland). Two orthopaedic surgeons with extensive experience in the software and blinded to the clinical data of each CT, identified the HSD and made the following measurements: the width and depth of the HSD was measured as proposed and validated by Ozaki et al. [17]: on an 3D en face view the most superolateral point and the most inferomedial point of the Hill-Sachs lesion was plotted, the line connecting the 2 points was defined as the major axis (the length of the lesion). Then, the longest line connecting the medial and lateral edges of the lesion perpendicular to the major axis was defined as the minor axis (the width of the lesion), then, on an axial CT image, a virtual circle that included the articular surface of the humeral head was drawn, and the depth of the Hill-Sachs lesion was defined as the longest length between the base of the lesion and the corresponding arc. These measurements were normalized dividing them by the diameter of the humeral head as recommended by Cho et al. [6] and Ozaki et al. [17]. To assess the volume of the humeral defect, the surface of each defect was marked in each axial slice using the Region Of Interest tool and the bone defect volume was calculated with a volumetric 3D reconstruction. All measurements were made twice, and the mean was used for further calculations. An increase of 25% in the depth, width or volume of the defect was arbitrarily considered as relevant.

The following clinical data were obtained for each participant: age, sex, mechanism of dislocation (high energy/low energy; traumatic or atraumatic), delay between dislocation and admission, delay between admission and first reduction attempt, duration of reduction manoeuvres, reduction method, and presence of associated neurovascular injuries.

No formal sample size calculation was performed. The study was designed as a pilot study to obtain preliminary data and a sample size of 20 was deemed appropriate. Due to the small sample size, the normality of the continuous variables could not be assumed, thus all statistical test used were non-parametric. The Chi-squared test was used to compare dichotomous and qualitative variables. The paired samples Wilcoxon test was used to compare quantitative variables. The statistical threshold for significance was stablished at *p* < 0.05.

This study was approved by the Institutional review Board of Hospital Universitario Ramón y Cajal (approval number 125–18; appendix 1). All subjects received oral and written information about the study and written consent for participation was obtained from all subjects included.

## Results

A total of 33 subjects with suspected first-time anterior shoulder dislocations were offered to participate in the study. Of these, 24 consented to participate and had the Pre-CT performed. In four of these an associated fracture was identified (two greater trochanter fractures, one surgical neck fracture and one anterior glenoid fracture affecting 30% of the glenoid surface) and were excluded from the study, leaving 20 subjects for assessment. All these had both the pre-CT and the Post-CT performed.

Twenty subjects, 13 males and 7 females, median (interquartile range) age 51.9(42.4) years, minimum 19 years, maximum 87 years, with 20 first-time anterior shoulder dislocations (14 right/ 6 left shoulders; 17 traumatic/ 3 atraumatic dislocations; 16 low-energy/ 4 high energy [motor vehicle accidents]) were included in the study. The median (interquartile range) delay between dislocation and admission was 225(180) min; the delay between admission and first reduction attempt was 89(121) min.

In 11 of 20 cases the traction distraction manoeuvre was used, the FARES technique was used in 5 shoulders and the Milch technique in four. These techniques were successful in 15 subjects (in the first attempt in 11 cases, in the second attempt in 4 cases) and required 15(23) min until reduction was obtained. Five subjects required sedation to attain closed reduction, this delayed reduction 41 to 450 min more. No subject presented any associated neurovascular injuries after reduction.

**Fig. 2:**
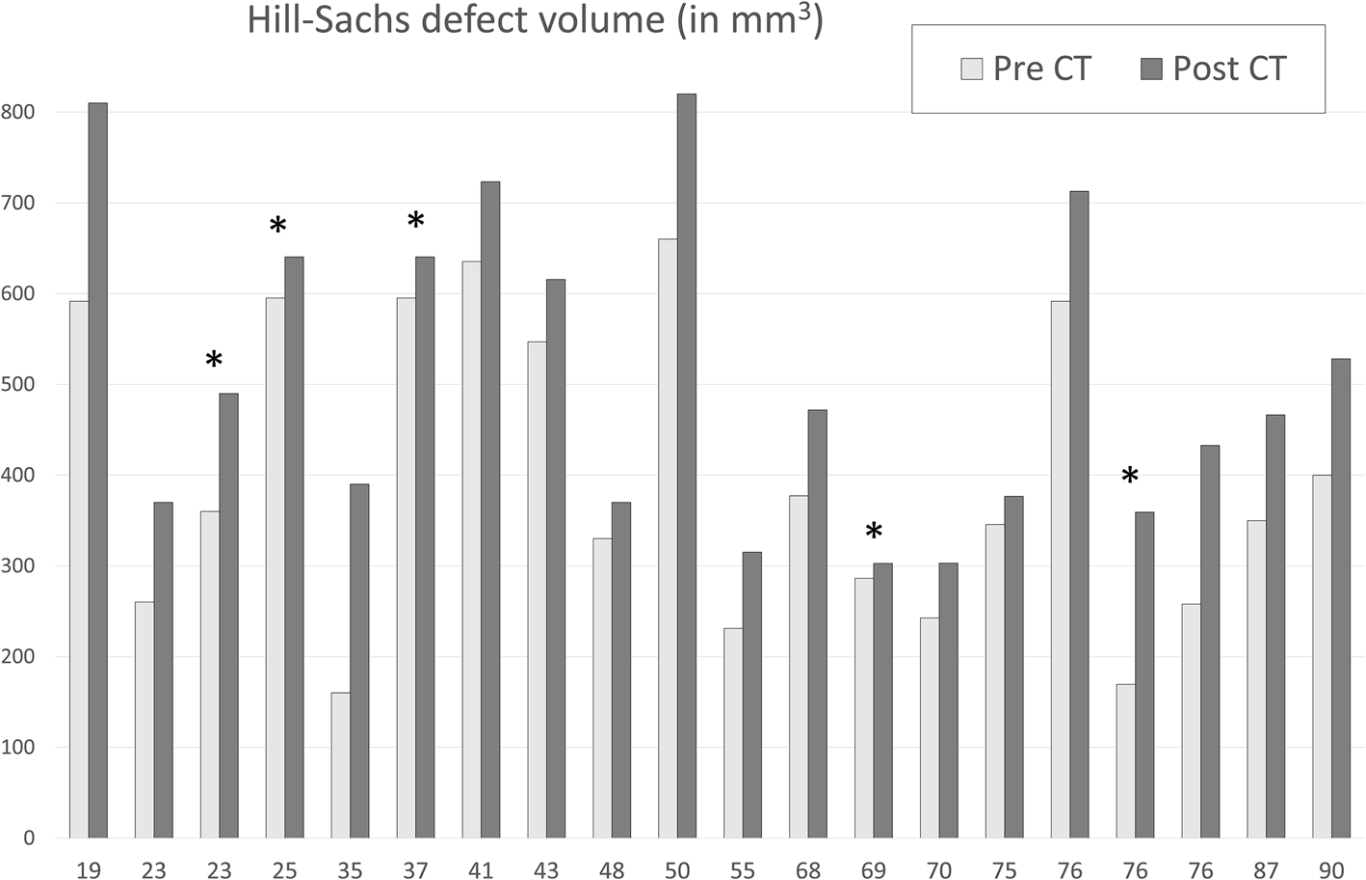
The volumes (in mm^3^) of the Hill Sachs Defects in the pre-reduction CT and post-reduction CT for all 20 cases. The cases are ordered in ascending order by age, the number in the x -axis is the age of each case. In 5 cases, marked with an asterisk (*), reduction was not achieved in the ED and required reduction under general anaesthesia

In all subjects an HSD was identified in the Pre-CT images. In 4 cases an anterior glenoid rim fracture (a bony Bankart) was identified. The post-CT scan were performed 8(26) days after the reduction. No further osseous lesions were identified, but the sizes (width, depth and volume) of the HSD increased in all lesions; these increases were significant (*p* < 0.001; Table 1, Fig. 2). The volume of the HSD increased more than 25% in 10 cases; in five cases there was an increase in the HSD depth larger than 25% and two subjects had increases in the HSD width of more than 25%. There were relevant increases in at least one these size parameters in 15 out of 20 subjects (75%).

**Table 1:** The median width, depth and volume of the defects as measured in the pre-reduction CT (Pre-CT) and post-reduction CT (Post-CT). The data is presented in absolute terms (in mm and mm^3^) and also in relative terms (divided by the diameter or volume of the head) in percentage form. All increases (Δ) were significant (*p* < 0.0001 for all variables and comparisons). All data is presented in median[interquartile range] form

	Absolute value		Values relative to ø		Δ (%)	*P* value
	Pre-CT	Post-CT	Pre-CT	Post-CT		
Width	9,9[2.9] mm	10.9[3] mm	28.8[10.1]%	30.3[10.6]%	7.23[8.48]%	*P* = 0.0001
Depth	7.0[3.0] mm	7.2[2.7] mm	16.9[9.8]%	17.3[9.5]%	9.93[20.7]%	*P* < 0.0001
Volume	355[333] mm^3^	469[271] mm^3^	4.4[4.4]%	5.8[3.3]%	25.0[24.6]%	*P* < 0.0001

A post hoc multivariate regression analysis evaluated the effect of age, number of reduction attempts, reduction manoeuvre and whether the dislocation had to be reduced under anaesthesia on the increase in size of the HSD. None of these factors significantly affected the HSD size, although the analysis was underpowered due to the small sample size.

## Discussion

The most important finding of this study is that, in this small sample of older adults with first time dislocations, the size of the HSD always increased during the reduction manoeuvre. This increase was larger than 25% in 15 of 20 cases. Thus, reduction manoeuvres without sedation in the ED may be effective in obtaining reduction but, in older adults, will increase the size of the HSD.

The HSD is probably the most frequent pathological finding in the anteriorly dislocated shoulder. In recurrent instability this lesion, in varying sizes, can be expected in 93 to 95% of cases [13, 25]. In subjects with first time dislocations the reported incidence in the literature varies more: Kim et al. [13] found, using magnetic resonance imaging, lesions in only 58% of cases after a first dislocation episode; Yiannakopoulos et al. [25] reported an incidence of 65% after arthroscopic exam of the shoulder, and Calandra et al. [5] found lesions during arthroscopy in only 47% of cases. Our results are not in line with these previous reports: we found measurable HSD lesions in the CT of all 20 cases; this high incidence is similar to that found by Owens et al. [16] who found HSD in 25 of 27 cases using MRI, and Taylor and Arciero [22] who found HSD during arthroscopic evaluation in 93% of 63 subjects with a fist-time anterior dislocation.

A proper assessment of the size and position of the HSD is essential to correctly evaluate the impact it has on the future instability on the shoulder. This was very early noted by Burkhart and Denard [4] and, more recently, the team of Yamamoto and Itoi have clarified its relevance when considering the glenoid track concept [8, 23, 24]. Despite its importance, appropriately measuring the size and position of the HSD is notably difficult and Schenider et al. [21]. found a variability in measurements of 19%. To avoid these issues in this study we followed closely the recommendations by Ozaki et al. [17] that have been shown to be very reproducible. Our lesions were slightly larger than those found by Ozaki et al. [17] that assessed 15 subjects with first time dislocations and found a mean depth of the HSDs of 7% of the diameter of the humeral head (15% in our study after reduction) and a mean width of 15% (30% in our study). This increased size might be related to the drastically different age of the population studied (20 years old in Ozaki´s study and 60 years old in ours). Our HSD are similar in size to those found in subjects with recurrent dislocations: Ozaki et al. [17] found mean widths of 10% and depths of 22% in subjects with two or more dislocations and Cho et al. [6] found mean widths of 52% and depths of 14%. Different studies seem to find very different values despite the uniformity of measurement systems suggesting a high variability.

The key finding in our study was that HSDs increase in size during the reduction of a shoulder dislocation. Our study suggest that the size of the HSD might increase at least 25% in four out of five subjects in which reduction is attempted without sedation in a typical ED setting. This increase might not be clinically relevant in some cases, but might have relevance in others, increasing the chances of recurrence. The HSD develops as the humeral head impacts with the strong anterior glenoid bone when the head is dislocated anteriorly. Kawakami et al. [12] have shown that the humeral defect shape resembles very clearly the shape and contour of the anterior glenoid rim; but, sometimes, the defect is slightly larger. They suggested that this increase in size was due to the movement of the arm, with the shoulder dislocated, as the patient tries to rotate the arm into a position of comfort. This was confirmed in a cadaveric study performed by the same group [9]. Our data suggest that part of the mishap between the glenoid shape and the HSD might be due to the damage done during reduction.

This study gives clear indication that an increase in the volume of the HSDs indeed happens during reduction. The presented data shows that the effect is large enough but should be followed with more research to find out if any specific group of subjects or an specific reduction manoeuvre has an increased risk of developing larger defects. We did not find any strong association of increased defect size in relation to age, reduction manoeuvre or repeated reduction attempts.

With these results in mind this study might have some practical implications: reductions attempts should not be done aggressively and it should be considered to be performed under sedation in older patients.

This study has some limitations. The first and foremost is the relative old age of the subjects included in the study. This is related to the very aged population our hospital takes care of and, probably to some degree of admission bias, as many first-time dislocations in younger subjects are reduced in-situ by paramedical personnel in sporting facilities or attending the injured. A potential recruitment bias might be possible, with younger subjects being less willing to take part in the study. Thus, our results can only be taken into account when considering the management of shoulder dislocation in older adults. The second is the small sample size, but this was expected as it was developed as a pilot study. We consider that the results found, strongly suggest that the HSDs increase in size during reduction, and another study, focusing in younger individuals and with a larger sample size is being considered. To finish, the reduction was initially attempted in the ED, without proper sedation and by relatively untrained physicians (orthopaedic surgery residents) that used different reduction methods; all this could imply that during the reduction manoeuvres more force than necessary might have been applied over a patient in significant pain, increasing the chances of progression of the HSDs or other fractures. This is clearly a limitation, but the setting and conditions presented here are similar to those present in many centres around the world in which these lesions are managed. Perhaps using strong sedation or general anaesthesia before any reduction attempt would have limited the damage to the humeral head. A trial that compares the bone lesions found after reduction with and without anaesthesia would help in elucidating this issue and is also being considered.

## Conclusions

Standard reduction manoeuvres performed in individuals with a first-time anterior shoulder dislocation increase the size of the HSDs. The HSD develops after the dislocation but can increase in size during reduction without sedation of the dislocated shoulder. This increase in size is larger than 25% in three out of four cases.

## Data Availability

All data is registered in Hospital Ramón y Cajal database.
